# Interobserver variability of myocardial T1 and T2 mapping in patients with suspected myocarditis

**DOI:** 10.1186/1532-429X-16-S1-P249

**Published:** 2014-01-16

**Authors:** Christian Luecke, William Rutschke, Clara Frank, Philipp Lurz, Matthias Grothoff, Ingo Eitel, Lukas Lehmkuhl, Holger Thiele, Matthias Gutberlet

**Affiliations:** 1 Diagnostic and Interventional Radiology, Heartcenter Leipzig, Leipzig, Germany; 2 Internal Medicine/Cardiology, Heartcenter Leipzig, Leipzig, Germany

## Background

The aim of this study was to test the interobserver variability of myocardial T1 and T2 Mapping prior to and T1 Mapping after the administration of contrast agent in patients with suspected myocarditis. The established methods for the detection of the presence of active inflammation - edema ratio (ER) and global relative enhancement (gRE) - require a "normal" reference region of interest (ROI) in adjacent skeletal muscle and depend on the choice of the muscle as well as its outline which can lead to high interobserver variability. Mapping techniques offer a quantitative approach without the need of a reference region.

## Methods

27 patients (17 male, 10 female; mean age, 49 ± 19 years) with clinically suspected myocarditis underwent Cardiovascular Magnetic Resonance Imaging (CMR) at 1.5 T (Gyroscan Intera CV, Philips Medical Systems, Best, the Netherlands). The CMR assessment included midventricular single slice T1 maps before (T1pre) and after (T1post) the administration of contrast agent (TR = 2,75-2,9 ms, TE = 1,38-1,45 ms, TI = 168-6659 ms, slice thickness = 10 mm, FOV = 380 × 380 mm, Matrix = 320 × 320) and T2 maps (T2) without contrast (TR = 545,45-1200 ms, TE = 11,66 - 48,27 ms, TI = 250 ms, slice thickness = 8 mm, FOV = 370 × 370 mm, Matrix = 384 × 384) in short axis orientation. Interobserver correlation and Bland-Altman-analysis was performed.

## Results

**Figure 1:**
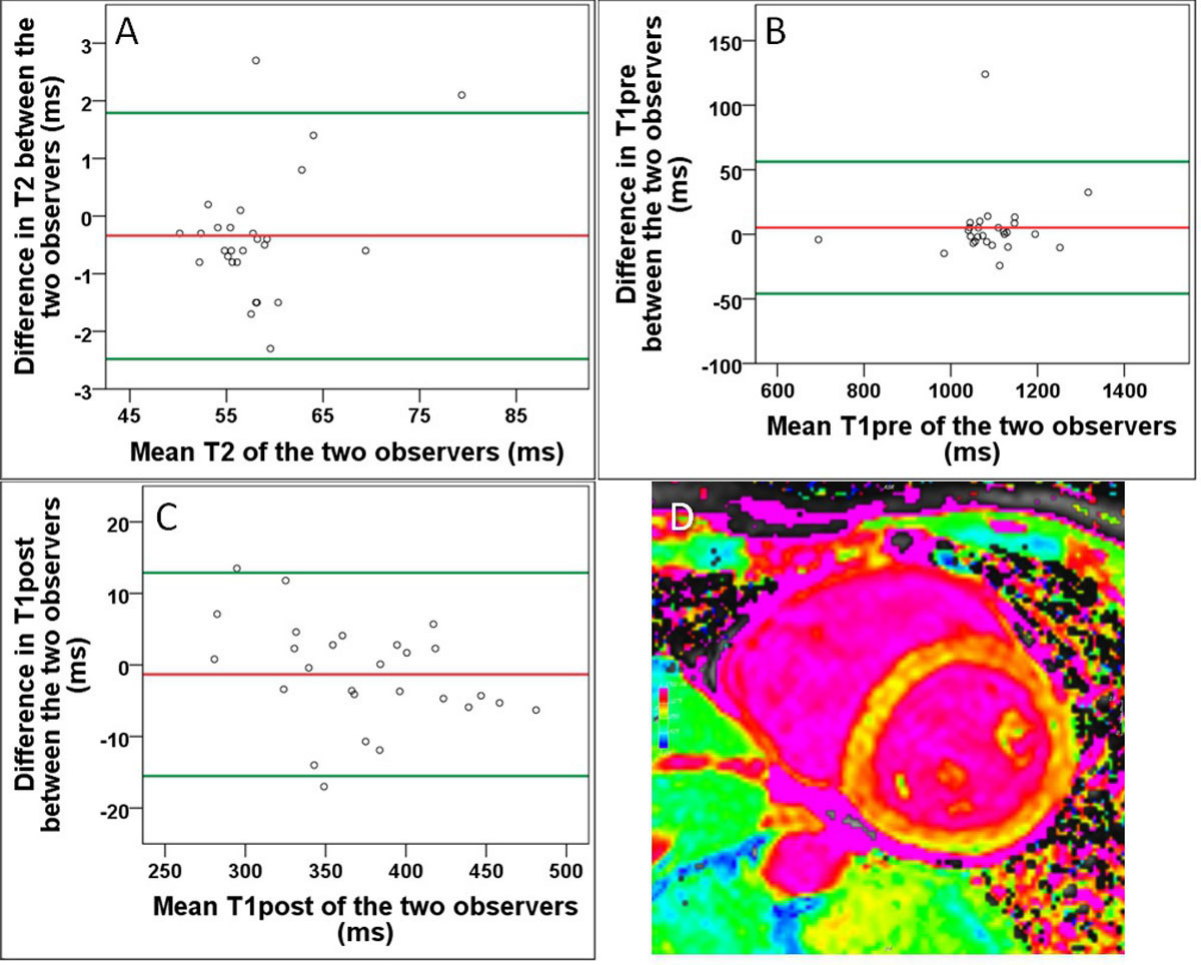
**Bland-Altman Analysis of global myocardial T2 (A) and T1 times before (B) and after (C) the administration of contrast agent**. Example of a T1 map before administration of contrast agent (D).

The interobserver agreement was high in T1pre, T1post and T2 mapping sequences (r = 0,97, r = 0,99 and r = 0,98, respectively) and showed only small interobserver bias (bias T1pre = 5,1 ms, bias T1post = -1,3 ms and bias T2 = -0,3 ms) with narrow limits of agreement (LOA T1pre = -45,9 and 56,2 ms, LOA T1post = -15,5 and 12,9 ms and LOA T2 = -2,5 ms and 1,8 ms, (Figure 1). The variation coefficient was lowest for T1post (9.5%) and T2 (9.9%) and low for for T1pre (14%).

## Conclusions

Myocardial T1 Mapping prior to and after the administration of contrast agent and myocardial T2 Mapping show very low interobserver variability and could be observer independent tools to evaluate patients with myocarditis by CMR. The relative limits of agreement were with 2% of the mean narrow for all three mapping sequences.

## Funding

None.

